# 

**Published:** 1902-08

**Authors:** 


### PERSONALS.

Dr. and Mrs. W. H. Hoskins of the JOURNAL made an extended trip in July through the West, visiting Helena, Montana, Yellowstone Park, Minneapolis, and St. Paul, Minnesota, Chicago, Ill., and other points.

Dr. J. P. Foster, formerly of Selby, South Dakota, has located at Beresford, South Dakota.

Dr. O. A. Stingley, of Kansas City, Missouri, has been appointed an assistant inspector in the Bureau of Animal Industry and ordered to Chicago.

Dr. Charles Ellis, who maintains a thoroughly up-to-date dog hospital in St. Louis, is having a dog ambulance constructed.

Drs. W. L. Rhoads and James Marshall, two of Eastern Pennsylvania's veterinarians, are members of the committee directing the Road Drivers' parade in Philadelphia in October.

Dr. Emil Knight, of Rochester, New York, is a great admirer and breeder of the English pointer dog. He only breeds from those bred in the blue.

Dr. D. B. Keeler, of Salem, Oregon, holds the position of Stock Inspector for Marion County.

Dr. Thomas Trinder, of Charlotte, N. C., will locate at Memphis, Tenn., after October 1st.

Dr. A. G. Kern, of Knoxville, Tenn., who graduated at the Veterinary Department, University of Pennsylvania, and afterward pursued a course in human medicine, is now attached to the staff of the German Hospital, Philadelphia, Pa.

Dr. A. L. Wood, formerly of Prairie City, Iowa, has located at Hampton, Iowa.

Dr. D. F. Luckey, State Veterinarian of Missouri, is a staunch advocate of legislation to regulate the practice of veterinary science in that State, in order that the stock owners may have the protection and assistance of properly qualified veterinarians.

Dr. Robert J. Morrison, of the Bureau of Animal Industry, has been transferred from East St. Louis to Chicago, Illinois.

Dr. Newton Lazarus, of Nazareth, Pa., has been afflicted with blindness and is bedfast. His practice has been sold to Dr. George L. Nicholas, a graduate of the Veterinary Department of the University of Pennsylvania, class of 1902.

Dr. W. Harry Lynch, graduate of the Kansas City Veterinary College, has located in Manchester, N. H. Dr. Lynch recently passed the examination of the New Hampshire State Board of Veterinary Medical Examiners.

Dr. Otto Noack, of Reading, Penna., President of the Schuylkill Valley Veterinary Association, has just undergone a serious cranial operation in Philadelphia.

Dr. N. B. Smith, of Kansas City, Kansas, is now located at Billings, Montana.

Dr. Alex. Glass, of Philadelphia, Pa., spent the month of July yachting. As a member of the Corinthian Yacht Club he was much interested in their yearly cruise in the waters of Long Island Sound.

Dr. James M. Mecray, of Maple Shade, N. J., is a frequent vistor to Atlantic City, and is a great lover of the surf.

Dr. W. H. Dalrymple's, of Baton Rouge, La., article in the *New Orleans Medical and Surgical Journal* on "The Value of Co-operation in the Sanitary Control of Our Periodic Epizootics of Anthrax," is reproduced in this number.

Dr. J. W. Vansant, graduate of the Veterinary Department of the University of Pennsylvania, class of 1902, has located at Fox Chase, Pa.

Dr. H. D. Martein, of Philadelphia, Pa., is sojourning at Ocean City, New Jersey, after a severe illness from poisoning, which confined him to the Chester Hospital for some time. His many friends will be glad to learn of his convalescence.

Dr. Harry Nunn, of Portland, Oregon, has removed to McMinnville, the same State. Dr. Nunn was formerly connected with the United States army.

Dr. J. C. Shaub, of Lancaster, Pa., has just met with the sad loss of his daughter, aged twenty-seven years, from blood poisoning following an operation.

Dr. James C. Corlies, of Newark, New Jersey, recently removed a twenty-five-cent piece of the date of 1898 from a horse's shoulder. The parts bore no evidence of any old scar by which it might have entered.

Dr. Levi Johnson, a graduate of the Veterinary Department of the University of Pennsylvania, is proprietor of the Oregon Veterinary Hospital.

Dr. A. A. Harmon, of Chelmsford, Mass., has located at Lowell, in the same State.

Dr. F. W. Huntington, of the port of Portland, Maine, holds the highest record for a single shipment of cattle. The vessel was sent forth with 1179 cattle. It required eighteen carloads of hay to provide for their needs in transit.

